# Supplementary figures and images for: BHLHE40 drives protective polyfunctional CD4 T cell differentiation in the female reproductive tract against Chlamydia
Source: PLoS Pathog. 2024 Jan 25;20(1):e1011983. doi: 10.1371/journal.ppat.1011983 (PMC10846703; doi:10.1371/journal.ppat.1011983)

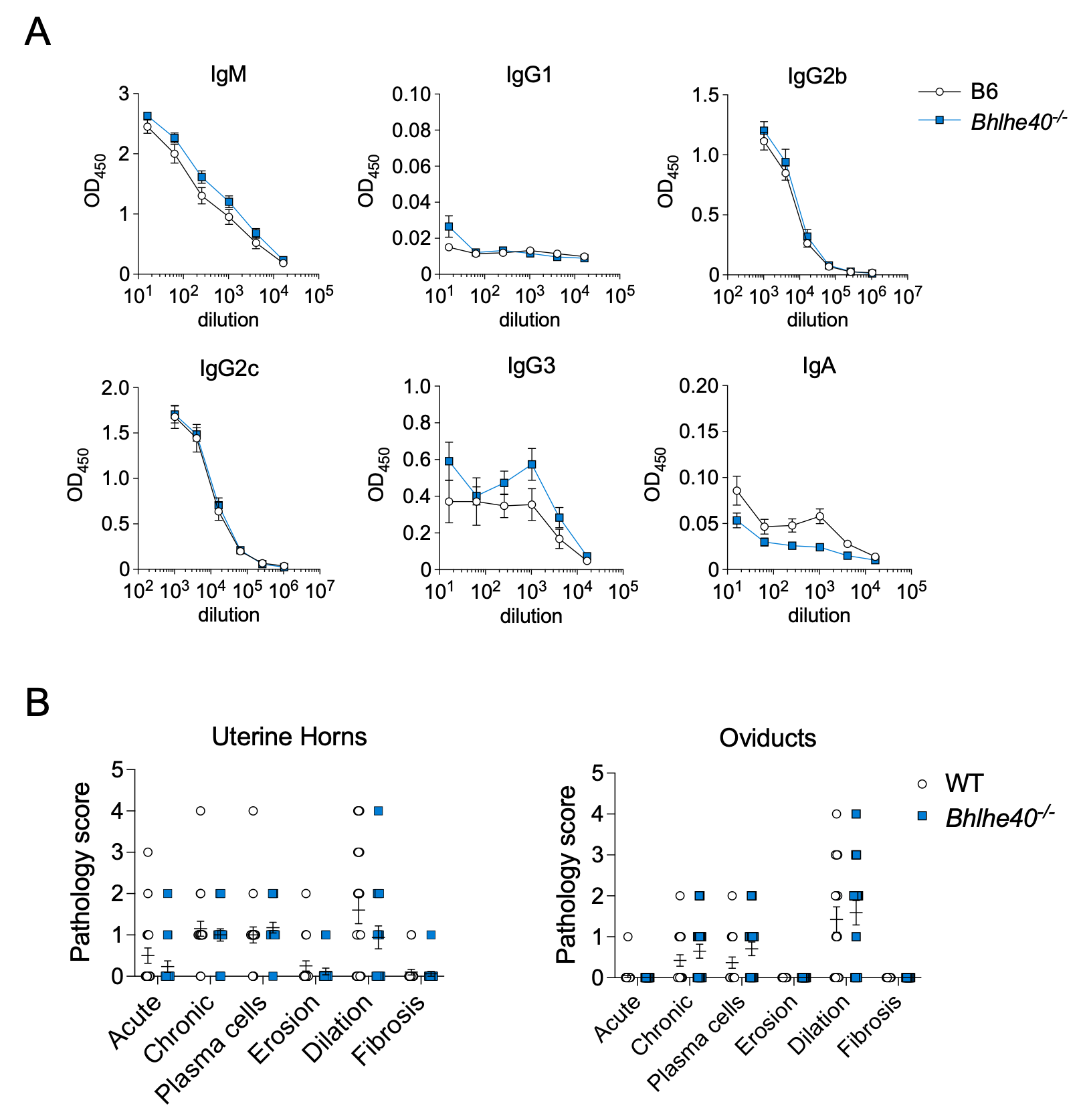

Supplement: S1 Fig — WT and Bhlhe40-/- mice were infected with 1×10^5^ C. muridarum. (A) Anti-Cm serum antibodies were measured at day 21 post infection by EB Ab ELISA. Data are from two independent experiments with 9 mice per group. Error bars represent the mean ± SEM. (B) FRTs were harvested between days 140–150 post infection. Pathology scores of uterine horns and oviducts were graphed for each category. Data are from two independent experiments with 8 to 10 mice per group. Each data point represents the left or right side of the corresponding section of the FRT. Error bars represent the mean ± SEM. (TIF) [file ppat.1011983.s001.tif]

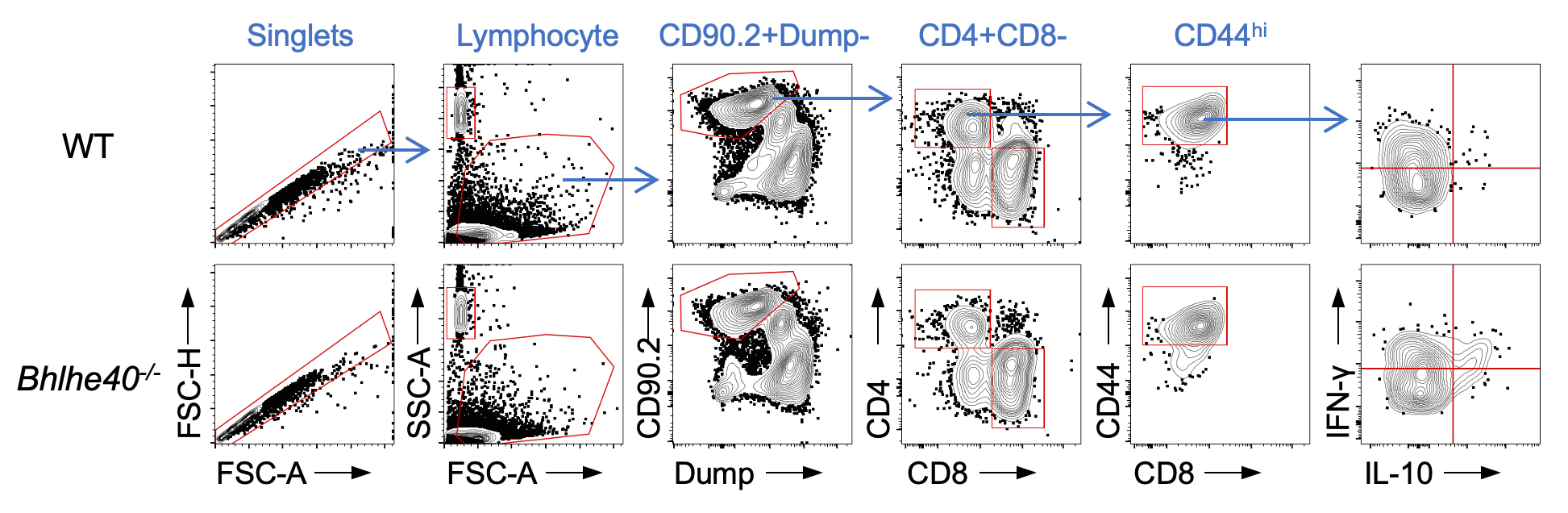

Supplement: S2 Fig — Representative FACS plots depicting gating strategy for activated CD4 T cells in the FRT of infected WT and Bhlhe40-/- mice (as in Fig 2A). Dead cells and unwanted cells were excluded using a Dump channel containing antibodies against CD11b, F4/80, B220 and FVD (fixable viability dye). (TIF) [file ppat.1011983.s002.tif]

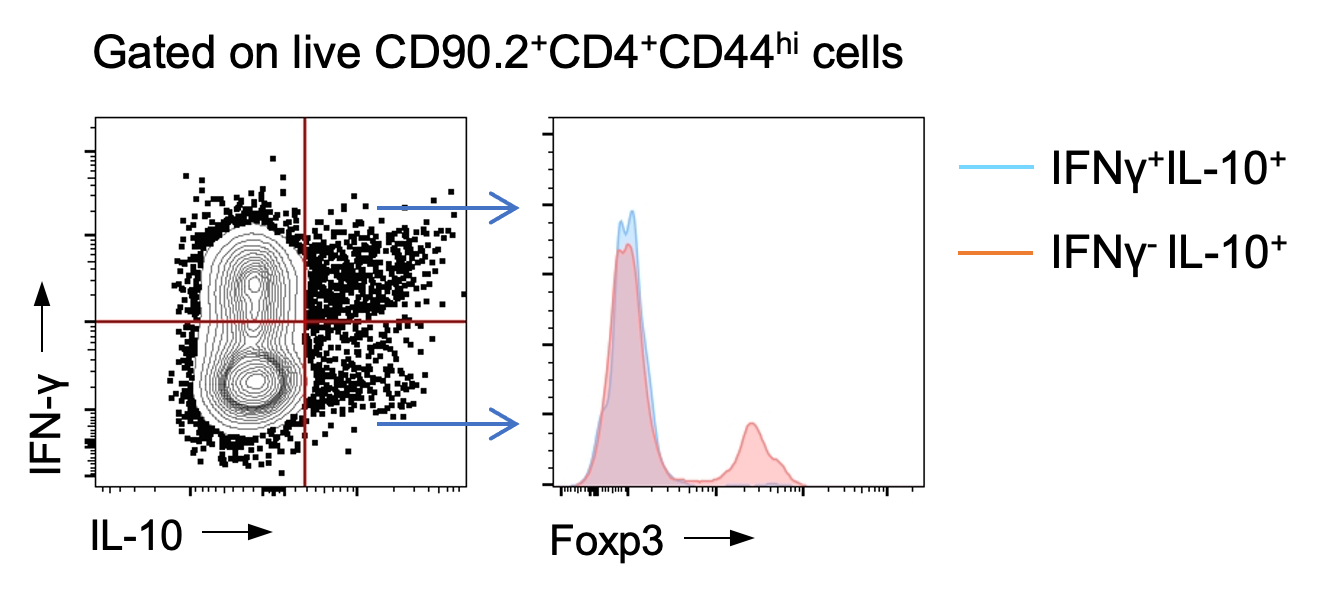

Supplement: S3 Fig — CD4 T cells from Bhlhe40-/- DLN samples in Fig 2A were analyzed for Foxp3 expression by intranuclear staining. Data are representative of two independent experiments. (TIF) [file ppat.1011983.s003.tif]
